# Localization of diacylglycerol lipase alpha and monoacylglycerol lipase during postnatal development of the rat retina

**DOI:** 10.3389/fnana.2014.00150

**Published:** 2014-12-15

**Authors:** Bruno Cécyre, Marjorie Monette, Liza Beudjekian, Christian Casanova, Jean-François Bouchard

**Affiliations:** ^1^Laboratoire de Neuropharmacologie, École d’Optométrie, Université de MontréalMontréal, QC, Canada; ^2^Laboratoire des Neurosciences de la vision, École d’Optométrie, Université de MontréalMontréal, QC, Canada

**Keywords:** endocannabinoid system, 2-arachidonoylglycerol, cannabinoid, immunohistochemistry, distribution, western blot, confocal microscopy, antibody

## Abstract

In recent decades, there has been increased interest in the physiological roles of the endocannabinoid (eCB) system and its receptors, the cannabinoid receptor types 1 (CB1R) and 2 (CB2R). Exposure to cannabinoids during development results in neurofunctional alterations, which implies that the eCB system is involved in the developmental processes of the brain. Because of their lipophilic nature, eCBs are synthesized on demand and are not stored in vesicles. Consequently, the enzymes responsible for their synthesis and degradation are key regulators of their physiological actions. Therefore, knowing the localization of these enzymes during development is crucial for a better understanding of the role played by eCBs during the formation of the central nervous system. In this study, we investigated the developmental protein localization of the synthesizing and catabolic enzymes of the principal eCB, 2-arachidonoylglycerol (2-AG) in the retinas of young and adult rats. The distribution of the enzymes responsible for the synthesis (DAGLα) and the degradation (MAGL) of 2-AG was determined for every retinal cell type from birth to adulthood. Our results indicate that DAGLα is present early in postnatal development. It is highly expressed in photoreceptor, horizontal, amacrine, and ganglion cells. MAGL appears later during the development of the retina and its presence is limited to amacrine and Müller cells. Overall, these results suggest that 2-AG is strongly present in early retinal development and might be involved in the regulation of the structural and functional maturation of the retina.

## Introduction

Cannabinoid receptors and their ligands constitute an endogenous signaling system that is particularly expressed in the CNS (Herkenham et al., 1991; Devane et al., 1992; Stella et al., 1997). This system is activated by exogenous cannabinoids such as Δ^9^-tetrahydrocannabinol (Δ^9^-THC) and is composed of cannabinoid receptors type 1 (CB1R) and 2 (CB2R), the endogenous agonists of these receptors, the endocannabinoids (eCBs) anandamide and 2-arachidonoylglycerol (2-AG), and the enzymes responsible for the biosynthesis and degradation of eCBs (Pertwee et al., 2010). The eCBs are modulators of synaptic transmission throughout the CNS. They act as retrograde inhibitors of neurotransmission and mediate the long-term depression of synaptic responses (Kreitzer and Regehr, 2001; Wilson and Nicoll, 2001; Sjöström et al., 2003). The levels of 2-AG in rodent brains are 800-fold higher than those of anandamide (Sugiura et al., 1995). Although anandamide binds to CB1R and CB2R with higher affinities than does 2-AG (Mackie et al., 1993), it acts only as a partial agonist, while 2-AG acts as a full agonist (Gonsiorek et al., 2000; Savinainen et al., 2001). On the basis of these differences between 2-AG and anandamide and on recent electrophysiological and anatomical studies, it is currently believed that 2-AG is the most potent natural ligand of cannabinoid receptors and is the key eCB for retrograde signaling in the brain (Sugiura et al., 2006; Katona and Freund, 2008; Tanimura et al., 2010). 2-AG is poorly soluble in the hydrophilic extracellular matrix (Devane et al., 1992; Di Marzo, 2008) and has a brief half-life (Járai et al., 2000). Consequently, its actions are local and dependent on the location of its biosynthesis site and receptors (Di Marzo, 2008). The synthesis of 2-AG is catalyzed primarily by diacylglycerol lipase alpha (DAGLα; Gao et al., 2010); 85% of its degradation is controlled by monoacylglycerol lipase (MAGL), and the enzymes alpha-beta hydrolase domain 6 (ABHD6) and 12 (ABHD12) hydrolyze the remaining 15% (Blankman et al., 2007).

The eCB system plays an important role in CNS development that includes neuron differentiation (Berghuis et al., 2005), neuronal migration, axon guidance (Argaw et al., 2011; Duff et al., 2013), positioning of cortical interneurons, morphogenesis and the specification and survival of neural progenitors (Guzmán et al., 2001; Galve-Roperh et al., 2006; see Harkany et al., 2007 for review). CB1R mRNA expression varies in the forebrain, brainstem and cerebellum during brain development (Berrendero et al., 1998). It has also been demonstrated that 2-AG levels progressively increase in the CNS throughout embryonic development, peak immediately after birth and stabilize during postnatal development (Berrendero et al., 1999; Fride, 2008).

Several constituents of the eCB system are located in the retinas of numerous species from fishes to primates (see Yazulla, 2008 for review). In adult rodents, CB1R, CB2R, DAGLα and MAGL are present in the retina (Hu et al., 2010; Zabouri et al., 2011a; Cécyre et al., 2013). To our knowledge, very few studies have examined the localization and/or effects of eCB biosynthesis and degradative enzymes in the developing retina. The consequences of CB1R modulation in the retinal cells of embryonic and young animals were studied by Lalonde et al. (2006) and Warrier and Wilson (2007). Briefly, these authors reported that CB1R activation modulates GABA release from amacrine cells and inhibits high-voltage-activated calcium channel currents in cultured ganglion cells, thereby affects the excitability of cells. Recently, our research group investigated the spatiotemporal distributions of CB1R and FAAH during postnatal development (Zabouri et al., 2011a,b). Our results demonstrated a differential distribution of CB1R and FAAH during this period. Apart from CB1R and FAAH, the expression and distribution patterns of retinal eCB system proteins during development remain unknown.

Because DAGLα and MAGL localization dictates 2-AG function, we investigated the spatiotemporal protein expression of DAGLα and MAGL in the rat retina during postnatal development until adulthood. The rodent retina represents a valuable model for the study of development because it includes several cell classes that comprise neuronal and glial cell types and because retinal cells have well-known developmental timelines (Rapaport et al., 2004). The results reported here show that DAGLα is constantly present during postnatal development. However, MAGL protein content is weak until postnatal day (P) 11, after which it increases until the adult age, and its distribution is not uniform throughout the retina. These results are consistent with the hypothesis that the eCB system is involved in the development of the retina during the postnatal period.

## Materials and methods

### Animals

All procedures were performed in accordance with the guidelines of the Canadian Council on Animal Care and the NIH guidelines for the care and use of laboratory animals, and were approved by the ethics committee on animal research of the Université de Montréal. Gestating adult Long-Evans rats were obtained from Charles River (St-Constant, QC) and maintained on a 12 h light/dark cycle. At least three pups from three litters were used at each age for the experiments.

### Tissue preparation

The rats were euthanized at various ages; i.e., P1, 3, 5, 7, 9, 11, 13, 15, 21, 30, 45 and as adults (≥P60). The animals were deeply anesthetized either by hypothermia (pups younger than P5) or with excess of isoflurane inhalation. One eye was immediately removed for western blot analysis. The retina was dissected on ice, promptly frozen and kept at −80°C until further processing. Simultaneously, a transcardiac perfusion was conducted with phosphate-buffered 0.9% saline (PBS; 0.1 M, pH 7.4) followed by phosphate-buffered 2% formaldehyde. The nasal part of the second eye was marked with a suture and removed. Two small holes were made in the cornea prior to an initial post-fixation in 2% formaldehyde for 1.5–2 h depending on the age of the animal (longer times were used for older animals). The cornea and lens were removed, and the eyecups were post-fixed for 10–30 min in 2% formaldehyde. The eyecups were washed in PBS, cryoprotected in 30% sucrose overnight, embedded in Neg 50 tissue Embedding Media (Fisher Scientific, Ottawa, ON), flash-frozen and kept at −80°C. Sections (14 µm thick) were cut with a cryostat (Leica Microsystems, Concord, ON) and placed on gelatin/chromium coated slides.

### Western blot

The retinas were homogenized in RIPA buffer (150 mM NaCl, 20 mM Tris, pH 8.0, 1% NP-40 (USB Corporation, Cleveland, OH, USA), 0.1% sodium dodecyl sulfate (SDS), 1mM EDTA), supplemented with a protease inhibitor mixture (aprotinin, leupeptin, pepstatin at 1 µg/ml and phenylmethylsulfonyl fluoride at 0.2 mg/ml; Roche Applied Science, Laval, QC). Thirty micrograms of protein/sample of the homogenate were resolved with 8 or 12% SDS-polyacrylamide gel electrophoresis (SDS-PAGE), transferred onto a nitrocellulose membrane, blocked with 5% skim milk and incubated overnight with antibodies directed against DAGLα, MAGL or β-actin, the latter served as a loading control. The blots were exposed to the appropriate HRP-coupled secondary antibodies (Jackson Immunoresearch Laboratories, West Grove, PA). Detection was performed using homemade ECL western blotting detection reagent (final concentrations: 2.5 mM luminol, 0.4 mM p-coumaric acid, 0.1 M Tris-HCl pH 8.5, 0.018% H_2_O_2_). The densitometric analyses were performed using ImageJ software (version 1.47b; Schneider et al., 2012) on scanned films obtained from a series of seven independent blots. Each analysis was performed with retinal samples from distinct experimental groups. The analyses were conducted following the recommendations of a number of reports (Tan and Ng, 2008; Gassmann et al., 2009). Briefly, a standard curve was produced from a serial dilution series to determine the linear dynamic range of detection of each antibody (Suzuki et al., 2011). Several exposure times were tested and only blots exposed below saturation were quantified. The DAGLα/β-actin and MAGL/β-actin ratios were calculated to compensate for the variability in the loading and concentration of the samples.

### Immunohistochemistry

Frozen sections were washed in PBS, post-fixed for 10 min in cold acetone, washed, and then blocked in 1% bovine serum albumin (Fisher Scientific, Ottawa, ON), bovine gelatin and 0.5% Triton X-100 in PBS for 1 h. The sections were incubated overnight in rabbit anti-DAGLα or anti-MAGL solution with an antibody against one of various retinal markers (see Table 1). The sections were subsequently washed in PBS, blocked for 30 min, and incubated for 1 h with the appropriate secondary antibodies, washed and mounted with PVA-Dabco mounting medium. Because antibodies to several retinal cell markers were raised in the same host as those for DAGLα and MAGL (recoverin, mouse cone-arrestin, calbindin, CtBP2 and VGlut1), these combinations required sequential incubations as previously described by our research group (Zabouri et al., 2011a,b) and others (Sherry et al., 2003). Briefly, the sections were labeled in a serial manner: the exposure to the first primary antibody was conducted as described above, followed by incubation in a goat anti-Fab fragment solution (Jackson Immunoresearch Laboratories). This allowed us to tag the first primary antibody as a goat rather than a rabbit. The sections were revealed with a secondary Alexa donkey anti-goat 633. The sections were then exposed to a second primary antibody overnight, and the latter was revealed with an Alexa donkey anti-rabbit 488 the following day. The dilution factors, immunogens, and provenance of the antibodies are provided in Table 1.

**Table 1:** **Antibodies used in this study**.

Antibody	Immunogen	Provenance	Dilution*	Host
DAGLα	C-terminus 42 amino acids of mouse DAGLα (1003–1044 amino acid residues); affinity purified with antigen polypeptide	DGLa-Rb-Af380, Frontier Institute, Ishikari, Hokkaido, Japan	I: 1/200W: 1/200	Rabbit
MAGL	N-terminus 35 amino acids of mouse MAGL (1–35 amino acid residues); affinity purified with antigen polypeptide	MGL-Rb-Af200, Frontier Institute	I: 1/200W: 1/200	Rabbit
β-actin	Modified β-cytoplasmic actin N-terminal peptide (DDDIAALVIDNGSGK, conjugated to KLH)	A5316, Sigma-Aldrich, St. Louis, MO	W: 1/2,000	Mouse
Mouse cone-arrestin (LUMIj)	C-terminus of the mCAR protein, residues 369–381; affinity purified with the immunogen	Dr. Cheryl M. Craft, Mary D. Allen Laboratory for Vision Research, Doheny Eye Institute, USC, Los Angeles, CA	I: 1/1000	Rabbit
Recoverin	Full-length recombinant human recoverin	AB5585, Millipore, Billerica, MA	I: 1/2,000	Rabbit
Calbindin	Recombinant rat calbindin D-28k full length	CB-38a, Swant, Bellinzona, Switzerland	I: 1/1,000	Rabbit
PKCα	Synthetic peptide with the sequence DFEGFSYVNPQFVHPILQSSV from the human protein	Sc-8393, Santa Cruz Biotechnology, Santa Cruz, CA	I: 1/500	Mouse
Syntaxin	Synaptosomal plasma fraction of rat hippocampus	S0664, Sigma-Aldrich	I: 1/500	Mouse
Brn-3a	Sequence: GGSAHPHPHMHGLGHLSHPAAAAAMNMPSGLPHPGLVAA fuzed to the T7 gene 10 protein	MAB1585, Millipore	I: 1/100	Mouse
Glutamine synthetase (GS)	Full protein purified from sheep brain	MAB302, Millipore	I: 1/3,000	Mouse
CtBP2	Amino acids 431–445 of rat CtBP2 coupled to keyhole limpet hemocyanin via added N-terminal Cys-residue; affinity purified with the immunogen	193003, Synaptic Systems, Göttingen, Germany	I: 1/10,000	Rabbit
PSD95	Recombinant rat PSD-95	MAB1596, Millipore	I: 1/200	Mouse
VGlut1	Synthetic peptide representing amino acids 456–560 of rat vesicular glutamate transporters (VGlut1); affinity purified with the immunogen	135303, Synaptic Systems	I: 1/10,000	Rabbit
MAP2	Bovine microtubule-associated protein 2	M1406, Sigma-Aldrich	I: 1/500	Mouse
PCNA	Synthetic peptide with the sequence LVFEAPNQEK	M0879, Dako, Burlington, ON, Canada	I: 1/500	Mouse
Alexa Fluor 488 donkey anti-mouse	Mouse (used against PKCα, syntaxin, Brn-3a, GS, PSD95, MAP2 and PCNA)	A-21202, Molecular Probes, Eugene, OR	I: 1/500	Donkey
Alexa Fluor 488 donkey anti-rabbit	Rabbit (used against mouse cone-arrestin, recoverin, calbindin, CtBP2 and VGlut1)	A-21206, Molecular Probes	I: 1/500	Donkey
Alexa Fluor 647 donkey anti-rabbit	Rabbit (used against DAGLα and MAGL)	A-31573, Molecular Probes	I: 1/500	Donkey
Alexa Fluor 647 donkey anti-goat	Goat (used against DAGLα and MAGL, for same hosts labeling)	A-21447, Molecular Probes	I: 1/500	Donkey
Peanut agglutinin (PNA)	No immunogen; binds to galactosyl (b-1,3) N-acetylgalactosamine, rhodamine labeled	RL-1072, Vector Laboratories, Burlingame, CA	I: 1/5,000	-

*I, immunohistochemistry; W, Western blot.

### Validity of the sequential labeling

The validity of the sequential labeling was verified for the rabbit-rabbit co-labeling with two controls. Briefly: (1) the second primary antibody was omitted, which yielded strong labeling with the goat secondary 633 and no labeling with the rabbit secondary 488; and (2) the first secondary and second primary antibodies were omitted, which yielded no signal for the goat secondary 633 and a low residual signal for the rabbit secondary 488. These control experiments demonstrated the specificity of the sequential Fab fragment protocol and were conducted systematically (data not show). Identical results have been presented in recent reports from our research group (Zabouri et al., 2011a,b).

### Antibody characterization

**Figure 1:**
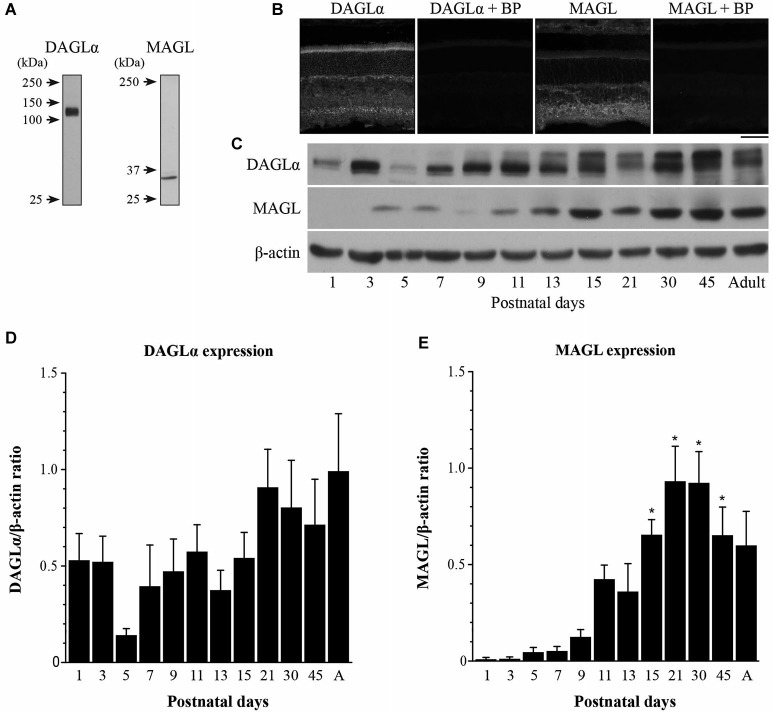
**Temporal patterns of protein contents of DAGLα and MAGL during retinal development. (A)** Characterization of the antibodies used against DAGLα and MAGL in the adult rat retina. Specific bands were seen at approximately 120 and 33-kDa for DAGLα and MAGL respectively. **(B)** DAGLα and MAGL expression in the adult rat retina in the presence or not of their respective blocking peptide (BP). Scale bar = 50 µm. **(C)** Representative examples of DAGLα and MAGL protein content during retinal development. **(D,E)** Average variations of DAGLα and MAGL during retinal postnatal development and maturation as measured by western blot analysis. Retinas were collected from rats between P1 and adulthood. The quantifications were performed on seven different sets of samples and mean optical density ratios ± SEM are presented for each age group. The statistical differences were assessed using a one-way ANOVA, Bonferroni *post hoc*-test. * Significant change compared to P1 (*P* < 0.05).

The DAGLα and MAGL antibodies have been fully characterized in the hippocampi of DAGLα and MAGL knockout mice (Yoshida et al., 2006; Uchigashima et al., 2011). Although DAGLα and MAGL share 100% homology in their protein sequence between rats and mice, we tested their specificities in the rat retina using western blotting. The anti-DAGLα and the anti-MAGL reacted with bands at approximately 120 and 33-kDa, respectively (Figure 1A), as described by other authors (Dinh et al., 2002; Yoshida et al., 2006; Suárez et al., 2008; Gao et al., 2010; Rivera et al., 2014). When the antibodies were pre-adsorbed with their respective blocking peptide, no immunoreactivity was detected in the adult rat retina (Figure 1B).

The mouse monoclonal antibody against beta-actin (β-actin) was raised against a slightly modified β-cytoplasmic actin N-terminal peptide (DDDIAAVIANGSGL). During western blotting, the β-actin antibody detects a single band at the expected molecular weight of 42 kDa (Gribaudo et al., 2009; Arellano et al., 2012).

The rabbit polyclonal antibody against mouse cone-arrestin was directed against the C-terminus epitope of the mCAR protein and detects a single band at 44 kDa in the mouse retina (Zhu et al., 2002, 2003; Nikonov et al., 2008). This antibody labels cone photoreceptors in the rat retina (Bobu et al., 2008; Bobu and Hicks, 2009).

The rabbit polyclonal antibody against recoverin was raised against recombinant human protein and recognizes a 25-kDa protein in the mouse retina. This calcium-binding protein has been detected in photoreceptors and cone bipolar cells of the retina, including the retinas of primates (Haverkamp and Wässle, 2000; Hendrickson et al., 2009) and rats (Milam et al., 1993; Chun et al., 1999; Günhan et al., 2003).

The rabbit polyclonal antibody against calbindin was directed against recombinant rat calbindin D-28K protein. This antibody detects a 28-kDa band on immunoblots and labels horizontal, ON cone bipolar cells and amacrine cell processes (Mitchell et al., 1995; Massey and Mills, 1996; Morgan et al., 2006; Hirano et al., 2011; Matsuoka et al., 2011). It labels horizontal cells (Pasteels et al., 1990; Peichl and González-Soriano, 1994) and amacrine cell processes in the rat retina (Mojumder et al., 2008).

The mouse monoclonal antibody against PKCα is specifically expressed in rod bipolar cells and dopaminergic amacrine cells (Negishi et al., 1988). Mouse anti-PKCα has been shown to exclusively recognize rod bipolar cells in the rat retina (Euler and Wässle, 1995; Johansson et al., 2000; Zabouri et al., 2011a). This antibody recognizes PKCα at approximately 80-kDa (Nagar et al., 2009). The immunoreactive pattern that we observed was similar to that reported in previous studies (Negishi et al., 1988; Gaillard et al., 2008).

The mouse monoclonal antibody against syntaxin-1 has been recognized as a specific marker of retinal amacrine cells by several research teams (Hirano et al., 2005; Li et al., 2010). Mouse anti-syntaxin recognizes syntaxin-1, which is a 35-kDa protein, in hippocampal, retinal and cortical neurons (Inoue et al., 1992). This antibody labels amacrine cells in the rat retina (Zabouri et al., 2011a; Kunzevitzky et al., 2013). The immunoreactive pattern observed in the present study was similar to that which has previously been reported (Li et al., 2004).

The mouse monoclonal antibody against Brn-3a labels only retinal ganglion cells in the retinas of several species (Gerrero et al., 1993; Xiang et al., 1995; Voinescu et al., 2009). According to the manufacturer, this antibody detects a 46-kDa band, does not recognize Brn-3b or Brn-3c, and does not label tissue from Brn-3a knockout mice. Brn-3a expression has been shown in the rat retina (Nadal-Nicolás et al., 2009; Zabouri et al., 2011a). Brn-3a labels the vast majority of ganglion cells but does not label intrinsically photosensitive retinal ganglion cells (ipRGCs; Jain et al., 2012).

The expression of glutamine synthetase (GS) in Müller cells has been demonstrated, and it has been established that the mouse monoclonal anti-GS labels a single 45-kDa band in adult rat retinal tissue (Chang et al., 2007). The labeling obtained with this antibody was comparable to that published elsewhere (Gargini et al., 2007; Kim et al., 2008).

The rabbit polyclonal antibody against the C-terminal binding protein 2 (CtBP2) recognizes ribbons in mammalian retina and produces a distinctive immunoreactivity pattern of horseshoe-shaped synaptic ribbons in the outer nuclear layer (ONL) and dense punctae in the inner plexiform layer (IPL). The antiserum recognizes a 110–120-kDa protein band and a 50-kDa protein band (tom Dieck et al., 2005), and has been tested in the rat retina (Østergaard et al., 2007).

The mouse monoclonal antibody to postsynaptic density protein 95 (PSD-95) detects a single band at approximately 100 kDa, which corresponds to the apparent molecular weight of PSD-95 on SDS-PAGE immunoblots from rat, mouse and bovine brains (manufacturer’s data sheet). The antibody also detects a minor band at approximately 75 kDa on western blots of mouse and rat brain extracts (manufacturer’s data sheet). In the rat retina, PSD-95 immunoreactivity is localized to photoreceptor terminals (Koulen et al., 1998; Li et al., 2012).

The rabbit polyclonal antibody against vesicular glutamate transporter 1 (VGlut1) was raised against a fusion protein containing amino acid residues 456–560 of rat VGlut1. This antibody recognizes a single 60-kDa band on western blots from the synaptic vesicle fraction of rat brains (Takamori et al., 2000). VGlut1 immunoreactivity is visible in photoreceptor and bipolar cell terminals of the rat retina (Johnson et al., 2007; Stella et al., 2008).

The mouse monoclonal antibody against microtubule-associated protein 2 (MAP2) recognizes a single 220-kDa band of rat brain (manufacturer’s data sheet) and specifically labels cell bodies and processes of ganglion cells in the rat retina (Okabe et al., 1989; Seki et al., 2003).

The mouse monoclonal antibody against proliferating cell nuclear antigen (PCNA) was chosen as a progenitor cell marker because it provides the least false positive and negative immunolabeling of progenitor cells in the retina (Barton and Levine, 2008). This antibody has been shown to label progenitor cell in the rat retina (Nakajima et al., 2001; Zabouri et al., 2011a). The specificity of this antibody has been fully characterized, and it recognizes a single band at 36 kDa (Waseem and Lane, 1990; Ino and Chiba, 2000; Pellegrini et al., 2007).

### Confocal microscopy

Images of the central retina (within 200 µm of the optic nerve head) were taken using a laser scanning confocal microscope (TCS SP2, Leica Microsystems) with 40X (NA: 1.25) or 100X (NA: 1.40) oil immersion objectives and 488 and 633 nm lasers. Image stacks (1,024 × 1,024 pixels × 0.5 µm per stack) were captured with a frame average of 3 using the LCS software (version 2.6.1; Leica Microsystems). Offline processing was performed with the Fiji software (version 1.47g; Schindelin et al., 2012). The stack images were taken sequentially and in distant wavelengths to ensure no “bleed-through” between channels. All images in which labeling intensities were compared were obtained under identical conditions of gain intensity. The Gaussian noise from the images was partially removed using the PureDenoise plugin for Fiji (Luisier and Blu, 2008), and the stacks were collapsed into projection images. Because gray scale photographs provide better contrast and more detail, individual channels are presented in gray scale, and the merged images are presented in color.

## Results

### Western blot analysis

The temporal patterns of expression of two elements of the eCB system in the retina were investigated by evaluating the total amounts of DAGLα and MAGL at various postnatal time points from P1 to adulthood. Representative examples of each enzyme are presented in Figure 1C. The lower blot displays the protein content of β-actin and demonstrates equal loading in all lanes. The averaged ratios of the optical densities are presented in Figures 1D,E, respectively. These proteins varied across development and exhibited different patterns. The DAGLα protein content was fairly stable from P1 to adulthood (no significant differences compared to P1, one-way ANOVA, Bonferroni *post hoc*-test, *p* < 0.05). In contrast, the amounts of MAGL protein were very low from P1 to P13, and then increased from P15 until P45 (P15, 21, 30 and 45 were all significantly different from P1 to P7, one-way ANOVA, Bonferroni post hoc-test, *p* < 0.05).

### Immunohistochemistry

**Figure 2:**
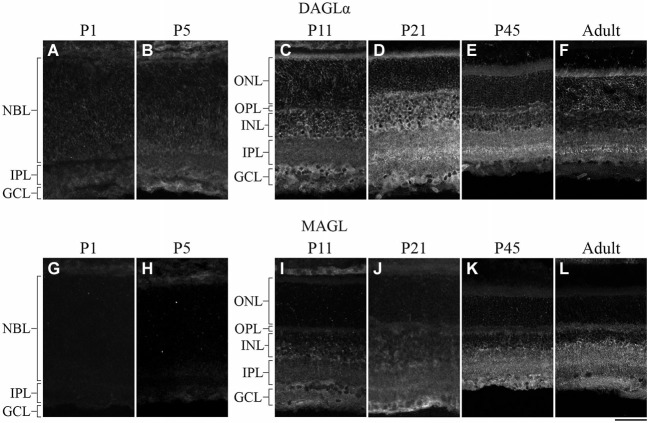
**DAGLα and MAGL immunoreactivity in the developing rat retina. (A–L)** DAGLα **(A–F)** and MAGL **(G–L)** protein expressions were obtained with vertical sections from P1, P5, P11, P21, P45 and adult retinas. GCL, ganglion cell layer; INL, inner nuclear layer; IPL, inner plexiform layer; NBL, neuroblast layer; ONL, outer nuclear layer; OPL, outer plexiform layer. Scale bar = 50 µm.

DAGLα was present in the neuroblast, inner plexiform and ganglion cell layers (NBL, IPL and GCL, respectively; Figure 2A) at P1. By P5, DAGLα immunoreactivity was largely upregulated, as indicated by the increased fluorescence exhibited by immunopositive cells compared to P1 (Figure 2B), which was present primarily in the GCL and in the inner nuclear layer (INL). At P11, DAGLα immunoreactivity was visible in the ONL, the outer plexiform layer (OPL), INL, IPL and GCL (Figure 2C). At P21, DAGLα localization remained similar (Figure 2D). This spatial profile was maintained over the following weeks (Figure 2E) until adulthood (Figure 2F) with increased fluorescence in the IPL. MAGL was not detectable in any neural layers of the retina at P1, but was weakly expressed exclusively in the retinal pigment epithelium (RPE; Figure 2G). At P5, MAGL immunoreactivity was barely visible in the RPE, the IPL and the GCL (Figure 2H). At P11, MAGL was present in the RPE, OPL, INL, IPL and GCL (Figure 2I). This expression pattern remained unchanged at P21, with the exception that MAGL was no longer present in the RPE (Figure 2J) over the following weeks (Figure 2K) until adulthood (Figure 2L).

### Cellular distribution of DAGLα and MAGL

#### Cone photoreceptors

##### DAGLα

**Figure 3:**
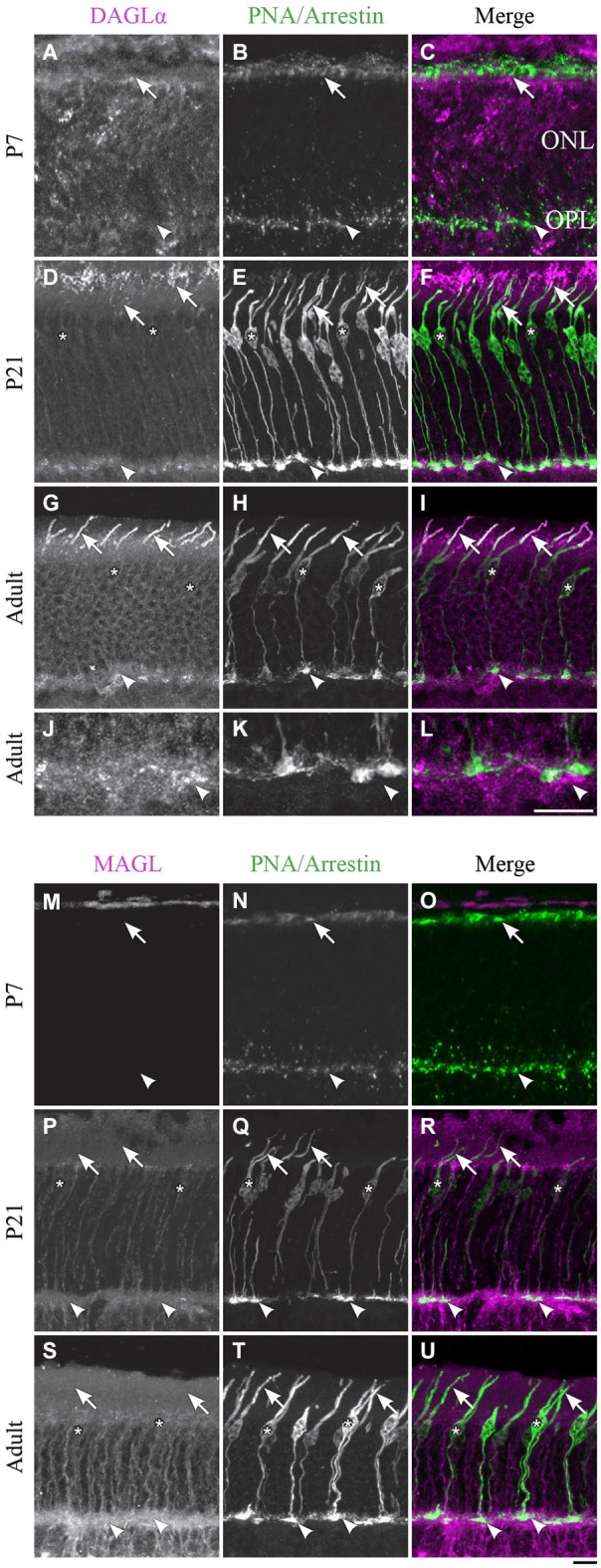
**DAGLα and MAGL immunoreactivity in cone photoreceptors. (A–U)** confocal micrographs of P7, P21 and adult rat retinas co-labeled for DAGLα **(A–L)** or MAGL **(M–U)** and the cell-type specific marker for cone photoreceptors, PNA (for P7) or cone-arrestin (for P21 and adult). Each protein is presented alone in gray scale: DAGLα or MAGL in the first column and PNA/cone-arrestin in the second; then the two are presented merged in the third column (DAGLα or MAGL in magenta and PNA/cone-arrestin in green). DAGLα is localized in the outer (arrows) and inner segments of cones, as well as the cell body (stars) but not the synaptic pedicle (arrowheads). MAGL is not detectable in any part of the cone photoreceptors. ONL, outer nuclear layer; OPL, outer plexiform layer. Scale bar = 10 µm.

Cones constitute the last class of early-born neurons to be generated (Rapaport et al., 2004). From P1 until P7, DAGLα was present in the outer segments but not in the pedicles of the cones (Figures 3A–C). By P21, the majority of cones reach their final position in the ONL, and they intensely expressed DAGLα in all of their cellular compartments except the cone pedicles (Figures 3D–F). Over the following weeks, DAGLα expression remained elevated in the outer and inner segments until the animal reached adulthood (Figures 3G–I). However, DAGLα was not expressed in the pedicles of the cones, but did seem to be present postsynaptically to these pedicles (Figures 3J–L).

##### MAGL

MAGL was not detectable in cone photoreceptors during retinal development in any of the cone cellular compartments (Figures 3M–U). MAGL expression in the ONL was exclusively localized to Müller cells (a detailed localization is presented in Figure 10).

**Figure 4:**
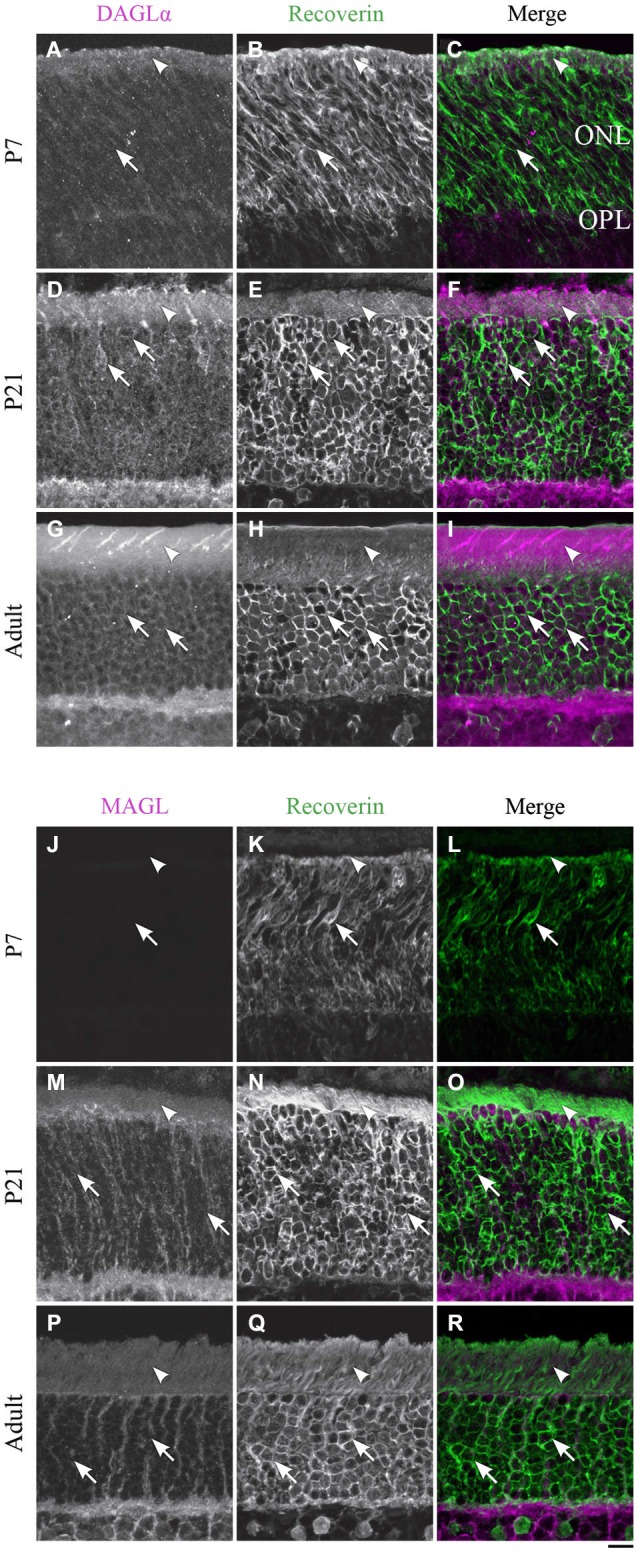
**DAGLα and MAGL immunoreactivity in rod photoreceptors. (A–R)** confocal micrographs of P7, P21 and adult rat retinas co-immunolabeled for DAGLα **(A–I)** or MAGL **(J–R)** and the cell-type marker for photoreceptors, recoverin. DAGLα is localized in the outer (arrowheads) and inner segments of the rods (arrows). MAGL is not detectable in any part of the rod photoreceptors. ONL, outer nuclear layer; OPL, outer plexiform layer. Scale bar = 10 µm.

**Figure 5:**
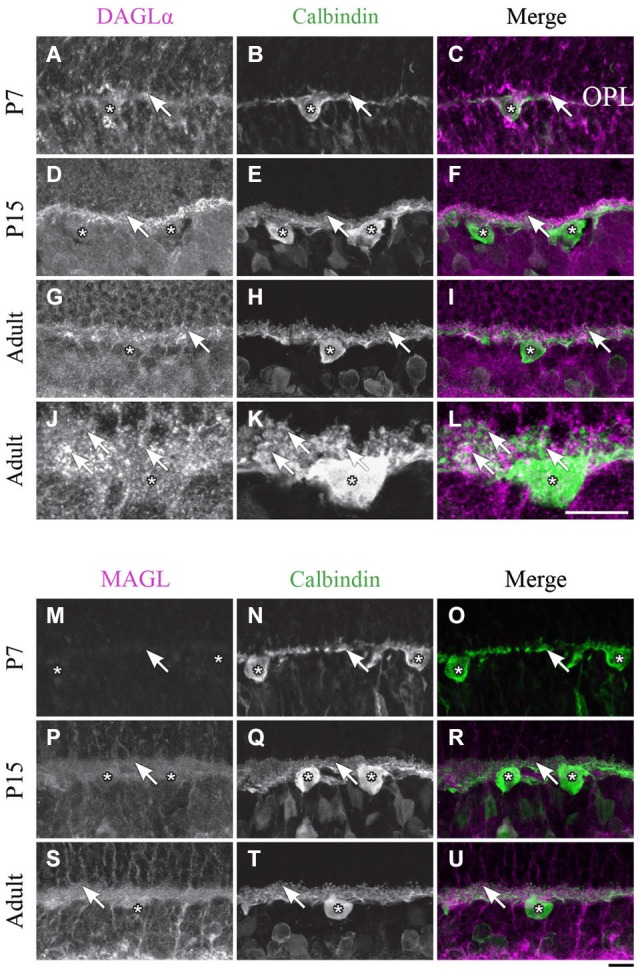
**DAGLα and MAGL immunoreactivity in horizontal cells. (A–U)** confocal micrographs of P7, P15 and adult rat retinas co-immunolabeled for DAGLα **(A–L)** or MAGL **(M–U)** and the cell-type specific marker for horizontal cells, calbindin. DAGLα is present in the dendritic terminals (arrows) but not in the cell bodies of the horizontal cells (stars). MAGL is not detectable in any part of the horizontal cells. OPL, outer plexiform layer. Scale bar = 10 µm.

**Figure 6:**
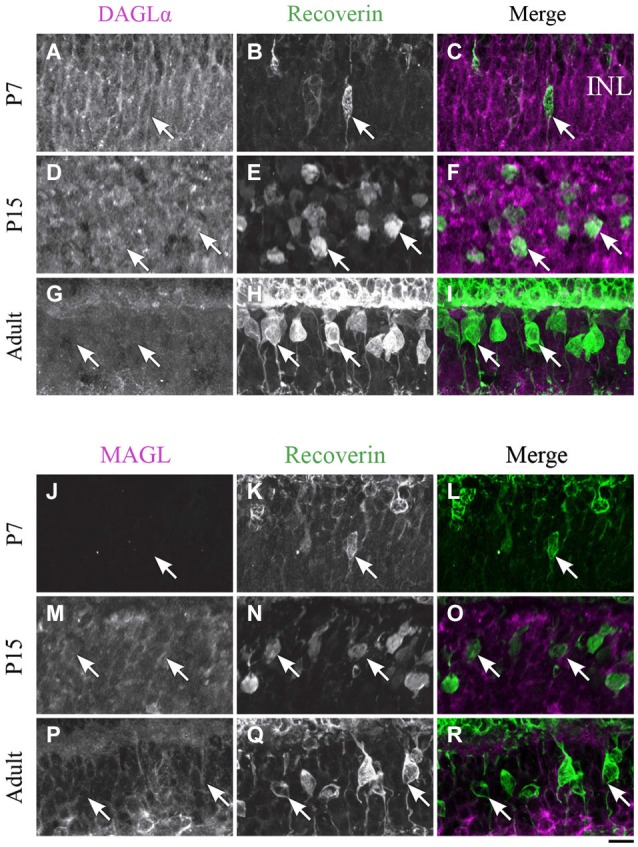
**DAGLα and MAGL immunoreactivity in type 2 and 8 cone bipolar cells. (A–R)** confocal micrographs of P7, P15 and adult rat retinas co-immunolabeled for DAGLα **(A–I)** or MAGL **(J–R)** and the cell-type marker for type 2 and 8 cone bipolar cells, recoverin. DAGLα is not expressed in the cell bodies (arrows) of type 2 or 8 cone bipolar cells. MAGL is not detectable in type 2 or 8 cone bipolar cells. INL, inner nuclear layer. Scale bar = 10 µm.

**Figure 7:**
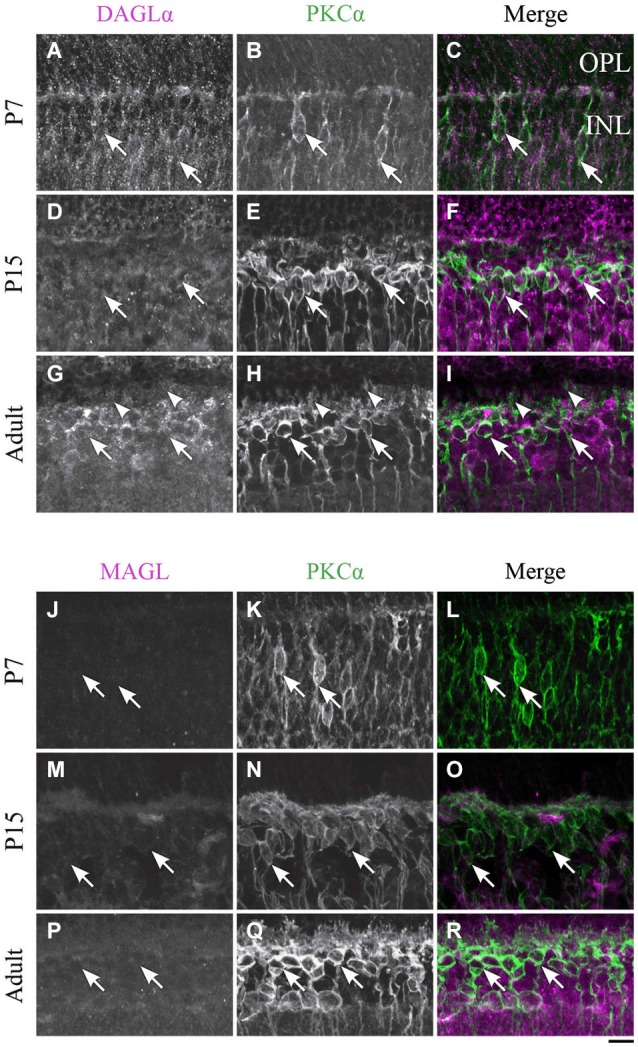
**DAGLα and MAGL immunoreactivity in rod bipolar cells. (A–R)** confocal micrographs of P7, P15 and adult rat retinas co-immunolabeled for DAGLα **(A–I)** or MAGL **(J–R)** and the cell-type specific marker for the rod bipolar cells, PKCα. DAGLα and MAGL are not detectable in the cell bodies (arrows) or dendritic connections of the rod bipolar cells. OPL, outer plexiform layer; INL, inner nuclear layer. Scale bar = 10 µm.

**Figure 8:**
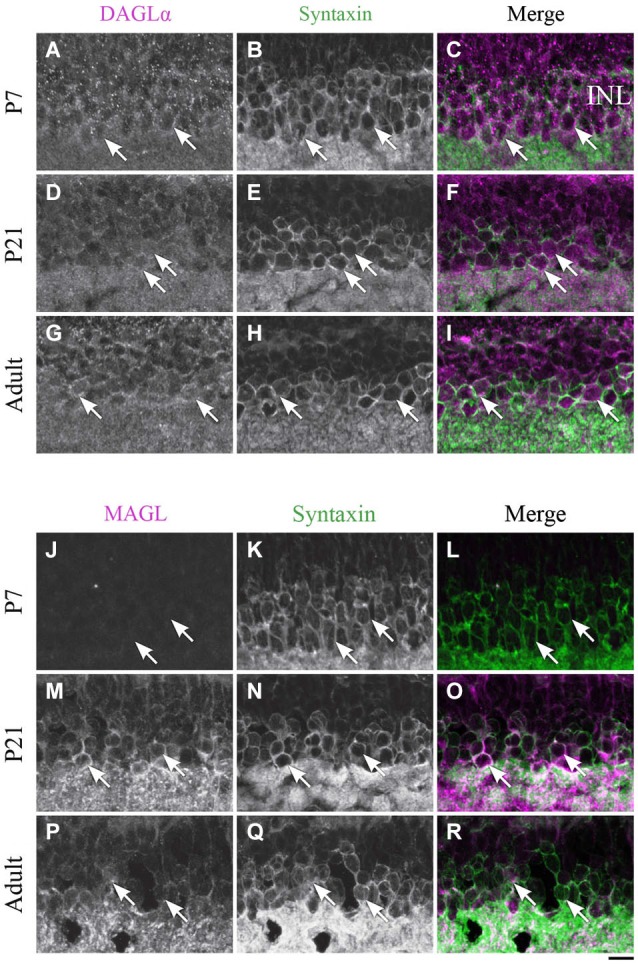
**Expression of DAGLα and MAGL in amacrine cells. (A–R)** confocal micrographs of P7, P21 and adult rat retinas co-immunolabeled for DAGLα **(A–I)** or MAGL **(J–R)** and the cell-type specific marker for the amacrine cells, syntaxin. DAGLα is localized in the cell bodies (arrows) of the amacrine cells from P1 to the adult age while MAGL is not detectable in these cells until P11. It is then expressed in the amacrine cells into adulthood. INL, inner nuclear layer. Scale bar = 10 µm.

**Figure 9:**
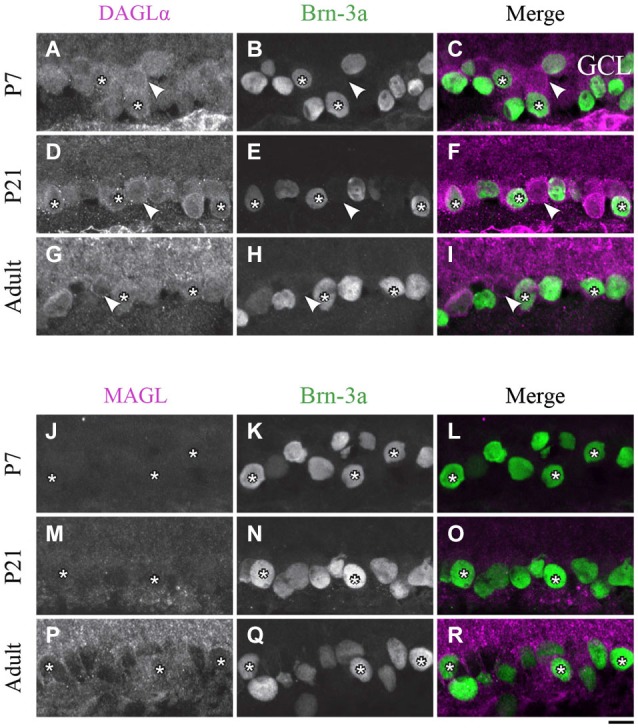
**DAGLα and MAGL immunoreactivity in ganglion cells. (A–R)** confocal micrographs of P7, P21 and adult rat retinas co-immunolabeled for DAGLα **(A–I)** or MAGL **(J–R)** and the cell-type specific marker for the ganglion cells, Brn-3a. DAGLα is localized in the cell bodies (stars) of the ganglion cells as well as in displaced amacrine cells or intrinsically photosensitive retinal ganglion cells (ipRGCs) (arrowheads) from P1 to the adult age. MAGL is not detectable in the ganglion cells. GCL, ganglion cell layer. Scale bar = 10 µm.

**Figure 10:**
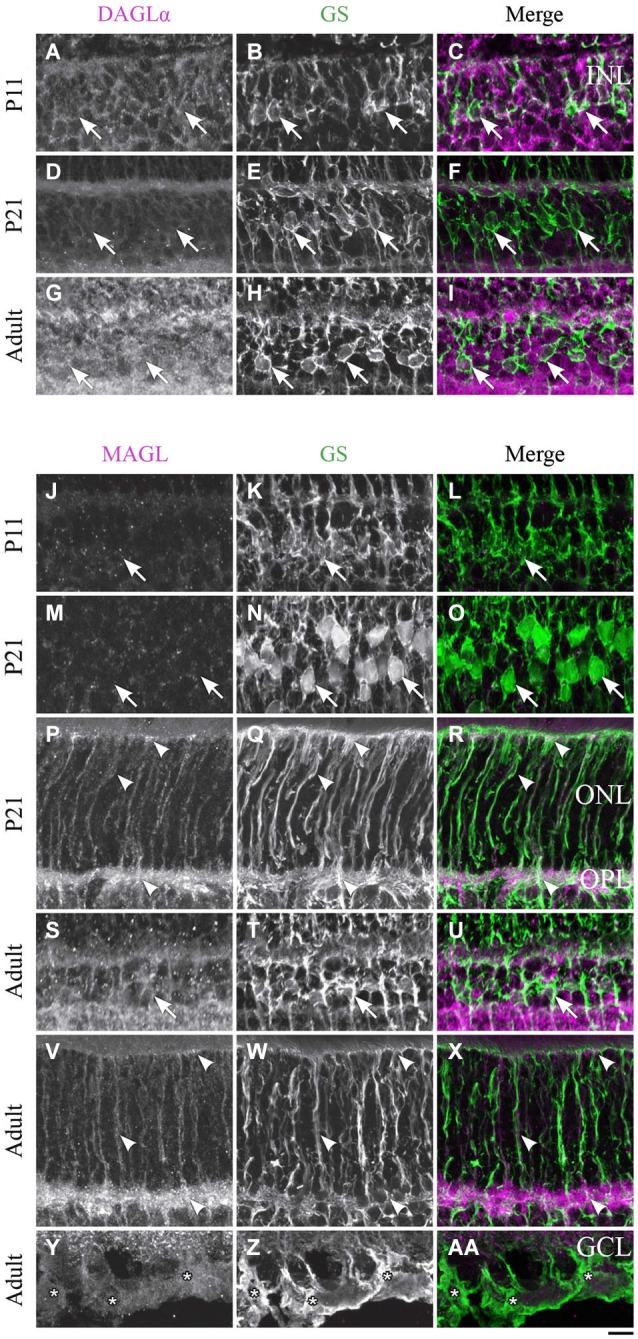
**DAGLα and MAGL immunoreactivity in Müller cells. (A–AA)** confocal micrographs of P11, P21 and adult rat retinas co-immunolabeled for DAGLα **(A–I)** or MAGL **(J–AA)** and the cell-type specific marker for the Müller cells, glutamine synthetase (GS). DAGLα is not expressed in the cell bodies (arrows), or any part of the Müller cells. MAGL is not detectable in the cell bodies of the Müller cells **(J–O, S–U)**. MAGL is localized in the outer **(P–R, V–X)** and inner **(Y–AA)** processes of the Müller cells from P11 to the adult age. INL, inner nuclear layer; ONL, outer nuclear layer; OPL, outer plexiform layer; GCL, ganglion cell layer. Scale bar = 10 µm.

#### Rod photoreceptors

##### DAGLα

Rods compose the vast majority of photoreceptors in the rat retina. In this study, the rods were discriminated from cones on the basis of their morphology. Starting from P1, DAGLα was expressed in somas of rods and in their inner and outer segments (Figures 4A–C). This expression remained constant through the second to the end of the third week of life (Figures 4D–F) and into adulthood (Figures 4G–I).

##### MAGL

MAGL was not detectable in any of the rod cellular compartments (Figures 4J–R). The immunoreactivity visible in the ONL was localized to Müller cells (Figure 10).

#### Horizontal cells

##### DAGLα

Starting from P1 and until P7, DAGLα expression was observed in the dendritic terminals of the horizontal cells (Figures 5A–C). This expression remained constant during the second and third weeks of life (Figures 5D–F), and until adulthood (Figures 5G–L).

##### MAGL

MAGL was not detectable in the OPL from P1 to P7 (Figures 5M–O). Starting from P11 and through to P45, light expression of MAGL was visible in the OPL but not in the horizontal cells (Figures 5P–R). This expression pattern was conserved in adulthood (Figures 5S–U).

#### Cone bipolar cells

##### DAGLα

Recoverin was also used as a marker to investigate DAGLα and MAGL expression in type 2 and 8 cone bipolar cells. This marker is present in type 2 OFF and type 8 ON cone bipolar cells. At P7, no recoverin-positive neurons expressed DAGLα (Figures 6A–C). The labeling of the somas remained the same from P15 (Figures 6D–F) to adulthood (Figures 6G–I), and neither type 2 nor 8 cone bipolar cells expressed DAGLα.

##### MAGL

MAGL was not detectable in recoverin neurons at P7 (Figures 6J–L). By P15, MAGL was still absent from cone bipolar cells (Figures 6M–O). MAGL remained undetectable in recoverin neurons by adulthood (Figures 6P–R).

#### Rod bipolar cells

##### DAGLα

Before P7, the majority of PKC-positive cells were either rod bipolar cells or amacrine cells. These cells could not be differentiated because most rod bipolar cells have not yet reached their final location at this stage. At P7, when the differentiation of the cells became clearer, DAGLα was absent from the somas of the rod bipolar cells (Figures 7A–C). The DAGLα signal remained similar at P15 (Figures 7D–F) and through adulthood (Figures 7G–I). Moreover, DAGLα was not expressed in the dendritic connections of rod bipolar cells.

##### MAGL

MAGL was not detectable in PKC neurons at P7 (Figures 7J–L). By P15, MAGL was still absent from rod bipolar cells (Figures 7M–O). This protein expression pattern continued into adulthood (Figures 7P–R).

#### Amacrine cells

##### DAGLα

DAGLα was expressed in amacrine cells during the first week of life (Figures 8A–C). All amacrine cells expressed DAGLα at P21 (Figures 8D–F) and through adulthood (Figures 8G–I).

##### MAGL

MAGL was not detectable in amacrine cells at P7 (Figures 8J–L), but an upregulation occurred from P11 (data not shown) and was also present at P21 (Figures 8M–O). MAGL was still expressed in amacrine cells in the adult animals (Figures 8P–R).

#### Ganglion cells

##### DAGLα

The spatial organization of DAGLα localization in ganglion cells did not vary with time. Examples of this expression are shown in Figures 9A–I. As expected, a decrease in the number of ganglion cells with age was observed. Moreover, GCL cells other than RGCs were also immunopositive; these cells might have been displaced amacrine cells or ipRGCs.

##### MAGL

At around P7, there was light expression of MAGL in the GCL but not in the ganglion cells (Figures 9J–L). From P21 to adulthood, the MAGL immunoreactivity intensity was elevated in the GCL but remained unobservable in the ganglion cells (Figures 9M–R).

#### Müller cells

##### DAGLα

Müller cells are generated late in development and begin to express GS at around P5. However, they are not clearly distinguishable until P11 and did not express DAGLα in their cell bodies or processes (Figures 10A–C). DAGLα was still undetectable in Müller cells from P21 (Figures 10D–F) to adulthood (Figures 10G–I).

##### MAGL

MAGL was expressed in the processes but not in the cell bodies of the Müller cells at P11 (Figures 10J–L). From P21, MAGL was present in the outer processes of Müller cells but remained absent from their cell bodies (Figures 10M–R). In adulthood, MAGL was expressed in the outer and inner processes of Müller cells but not in their cell bodies (Figures 10S–AA).

#### Outer plexiform layer

##### DAGLα

**Figure 11:**
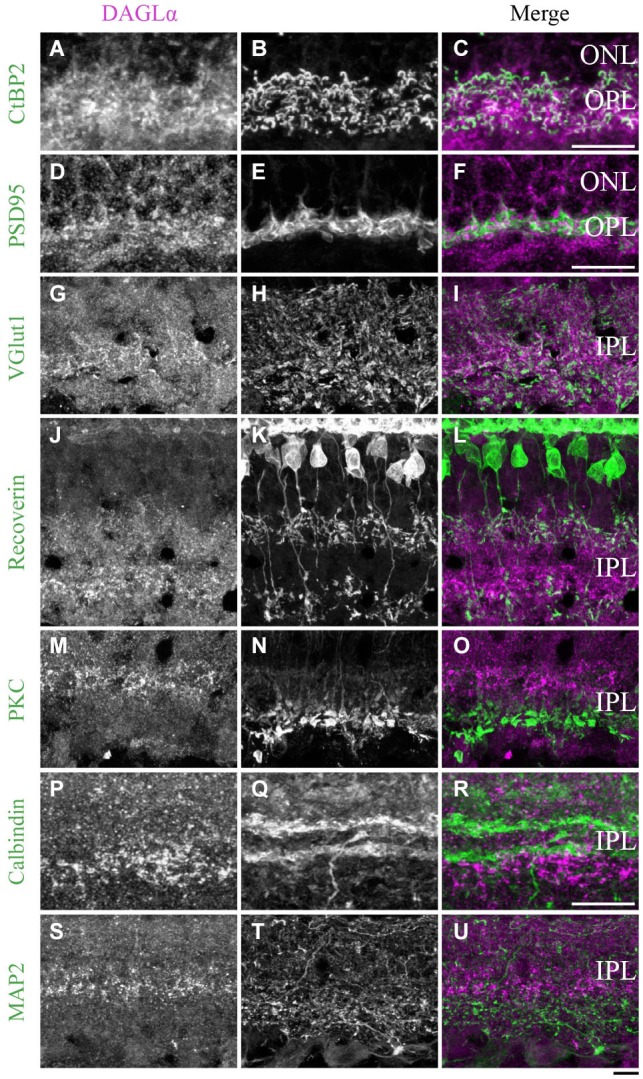
**DAGLα immunoreactivity in the outer and inner plexiform layers. (A–U)** confocal micrographs of adult retinas co-immunolabeled for DAGLα and several markers for photoreceptor synaptic ribbons (CtBP2), photoreceptor terminals (PSD95), axon terminals of the bipolar cells (VGlut1), axon terminals of type 2 and 8 cone bipolar cells (recoverin), axon terminals of rod bipolar cells (PKCα), dendrites of amacrine cells (calbindin) or dendrites of ganglion cells (MAP2). DAGLα is localized in the synaptic terminals of the photoreceptors **(A–F)**, and in the axon terminals of the bipolar cells **(G–I)**. However, DAGLα is not expressed in the axon terminals of type 2 and 8 cone bipolar cells **(J–L)** or rod bipolar cells **(M–O)**. DAGLα is not expressed in the dendrites of amacrine **(P–R)** or ganglion cells **(S–U)**. ONL, outer nuclear layer; OPL, outer plexiform layer; IPL, inner plexiform layer. Scale bar = 10 µm.

In the previous paragraphs, DAGLα was shown to be located in the OPL, specifically in the dendritic terminals of the horizontal cells (Figures 5A–L) and not in the pedicles of the cones (Figures 3J–L) or the dendritic connections of the rod bipolar cells (Figures 7G–I). We carefully examined whether this OPL labeling was exclusive to the horizontal cells. From P7 to adulthood, DAGLα was also expressed in the cytoplasm of the photoreceptor terminals (Figures 11A–F).

#### Inner plexiform layer

##### DAGLα

During development, strong DAGLα labeling was visible in the IPL. The antibody VGlut1 revealed the presence of DAGLα in the axon terminals of the bipolar cells (Figures 11G–I). Using the antibody recoverin, we determined that DAGLα was not expressed in the axon terminals of the type 2 or 8 cone bipolar cells (Figures 11J–L). The antibody PKCα revealed that DAGLα was not present in the axon terminals of the rod bipolar cells (Figures 11M–O). Finally, the antibodies calbindin and MAP2 confirmed that DAGLα was absent from the dendrites of the amacrine (Figures 11P–R) and ganglion cells (Figures 11S–U), respectively.

##### MAGL

**Figure 12:**
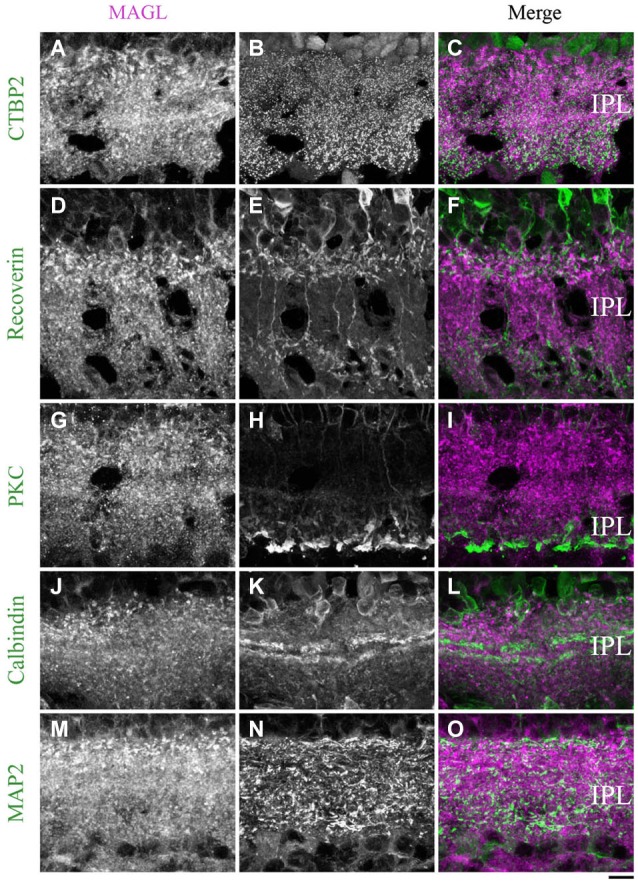
**MAGL immunoreactivity in the inner plexiform layer. (A–L)** confocal micrographs of adult retinas co-immunolabeled for MAGL and several markers for axon terminals of bipolar cells (CtBP2), axon terminals of type 2 and 8 cone bipolar cells (recoverin), axon terminals of rod bipolar cells (PKCα) or dendrites of ganglion cells (MAP2). MAGL is localized in the axon terminals of bipolar cells **(A–C)**, but not in the axon terminals of type 2 and 8 cone bipolar cells **(D–F)** or rod bipolar cells **(G–I)**. MAGL is not expressed in the dendrites of amacrine **(J–L)** or ganglion cells **(M–O)**. IPL, inner plexiform layer. Scale bar = 10 µm.

From P21 to adulthood, a MAGL labeling was visible in the IPL. The antibody CtBP2 revealed the presence of MAGL in the axon terminals of bipolar cells (Figures 12A–C). We then determined that MAGL appeared to be present in the axon terminals of the type 2 OFF but not in the type 8 ON cone bipolar cells (Figures 12D–F). The antibody PKCα revealed that MAGL was not present in the axon terminals of the rod bipolar cells (Figures 12G–I). Finally, the antibodies calbindin and MAP2 confirmed that MAGL was not expressed in the dendrites of the amacrine (Figures 12J–L) or ganglion cells (Figures 12M–O).

#### Progenitor cells

##### DAGLα

**Figure 13:**
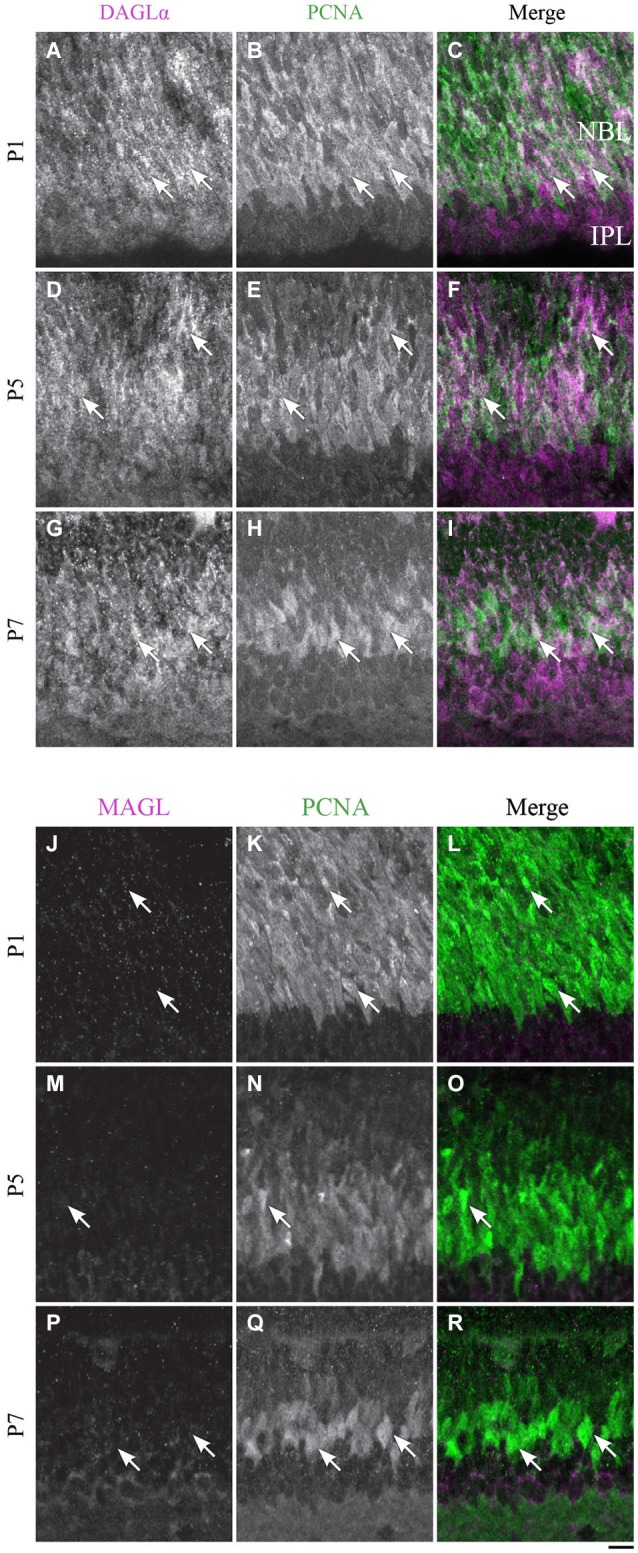
**DAGLα and MAGL immunoreactivity in progenitor cells. (A–R)** confocal micrographs of P1, P5 and P7 rat retinas co-immunolabeled for DAGLα **(A–I)** or MAGL **(J–R)** and the cell-type specific marker for the progenitor cells, PCNA. DAGLα is localized in the cell bodies (arrows) of progenitor cells from P1 to P7, while MAGL is not detectable in them. NBL, neuroblast layer; IPL, inner plexiform layer. Scale bar = 10 µm.

At P1, the progenitor cells were DAGLα-positive (Figures 13A–C), and the same pattern of expression was visible at P5 (Figures 13D–F) and P7 (Figures 13G–I).

##### MAGL

MAGL was not detectable in the progenitor cells at P1 (Figures 13J–L), and this pattern remained unchanged at P5 and P7 (Figures 13M–R).

#### Comparison of the temporal and spatial profiles of DAGLα and MAGL protein localization

**Figure 14:**
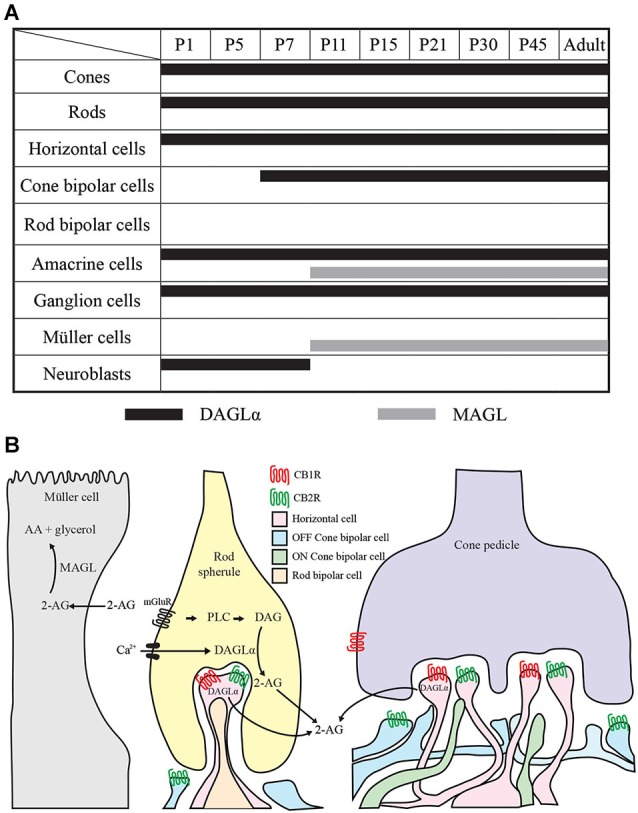
**Comparison of DAGLα and MAGL retinal localization. (A)** summary of the retinal localization of DAGLα (black bars) and MAGL (gray bars) over the postnatal development. **(B)** schematic illustration representing the localization and the hypothetical mechanism of action on synapses in the adult rat retina. The hydrolysis of membrane phospholipids into diacylglycerol (DAG) by phospholipase C (PLC) is promoted by the activation of G_q/11_ –coupled metabotropic receptors (mGluR) and/or by intracellular Ca^2+^ elevation. DAG is then conversed into 2-AG by DAGLα. 2-AG can bind to CB1R and/or CB2R present on retinal cells. 2-AG is later catabolized to arachidonic acid (AA) and glycerol by the enzyme MAGL present in the Müller cells. The panel B is partly based on results from Zabouri et al. (2011a) and Cécyre et al. (2013).

The timelines of DAGLα and MAGL localization in all of the retinal cells studied are shown in Figure 14A. These timelines were constructed based on detailed analyses of the expressions of both proteins in each cell type. From this figure, it can be seen that, during the first week of life, DAGLα was highly expressed in every cell type, while MAGL was not present in any of these cells. From P11, the two proteins were only co-expressed in amacrine cells. MAGL was present in the Müller cells from P11, while DAGLα was never expressed in these cells. In the adult retina, DAGLα and MAGL were only co-expressed in the amacrine cells. Hypothetical mechanism of 2-AG action on the retinal synapses of adult animals is presented in Figure 14B.

## Discussion

This study is the first to investigate the protein localization of DAGLα and MAGL, which are two key enzymes of the eCB system, during the postnatal development of the retina. Using immunohistological labeling and immunoblots, we showed that the protein content of DAGLα remains constant after birth, while that of MAGL varies during postnatal development and the maturation of the rat retina. Our data provide evidence of the early and widespread presence of DAGLα.

### Temporal expression of DAGLα and MAGL

We observed that the amount of DAGLα protein was constant during retinal development. These results are in agreement with previous reports that have suggested that DAGLα protein levels remain stable during brain development (for review, see Anavi-Goffer and Mulder, 2009). Conversely, the MAGL protein content was low from P1 to P13 and subsequently gradually increased from P15 to P45. Based on these results, we hypothesize that 2-AG levels are elevated from birth to P13 and are then regularized from P15 to adulthood. Hence, these results are in accordance with those of previous reports that have shown rapid increases in 2-AG levels immediately after the birth that are followed by stabilization during postnatal development (Berrendero et al., 1999; Fride, 2008).

### Cellular localization of DAGLα and MAGL

#### Cellular localization of DAGLα

DAGLα was present in the majority of early-born neurons as early as P1. This finding is in accordance with previous reports that have shown that DAGLα is expressed in the rostral migratory stream of young mice (Oudin et al., 2011). Hence, in the adult, we showed that DAGLα was present in cone and rod photoreceptors, horizontal cell processes, some cone bipolar cell axon terminals, amacrine cells, and ganglion cells. This expression pattern contrasts with that previously described for the mouse retina (Hu et al., 2010) in which DAGLα is present in postsynaptic terminals of type 1 OFF cone bipolar cells and is widely and diffusely distributed in the IPL. However, these findings are not in complete opposition with ours because we noted strong labeling in the OPL, which might correspond to the dendritic connections of cone bipolar cells, and in the IPL. This difference might be attributable to species differences in DAGLα expression between rats and mice.

#### DAGLα localization in the OPL

DAGLα was intensely expressed in the OPL; it was expressed in photoreceptor terminals but not in cone pedicles. Logically, these findings indicate that DAGLα is expressed in rod photoreceptor terminals. Moreover, we also demonstrated that DAGLα is expressed in the processes of horizontal cells but not in the dendritic connections of rod bipolar cells. We were unable to verify the presence of DAGLα in the dendritic connections of cone bipolar cells. Therefore, in the OPL, DAGLα is present in rod photoreceptor terminals, horizontal cell processes and possibly in the dendritic connections of cone bipolar cells.

#### DAGLα localization in the IPL

DAGLα is also intensely expressed in the IPL. We showed that DAGLα was not expressed in the dendrites of amacrine or ganglion cells. We also determined that DAGLα was present in the axon terminals of bipolar cells, but these connections were not from type 2 or 8 cone bipolar cells or rod bipolar cells. Comparisons of the shapes and positions of DAGLα labeling to those of the axon terminals of type 2 and 8 cone bipolar cells, as shown in Figures 11J–L, allowed for a hypothesis about the cell types in which DAGLα was present. Based on Figure 8 of Ghosh et al. (2004), which shows the stratification of each type of bipolar cell in the rat retina, we propose that DAGLα is expressed in the axon terminals of type 6 and/or 7 ON cone bipolar cells.

#### Cellular localization of MAGL

MAGL was minimally expressed in the retina until P11. At adulthood, MAGL was expressed in amacrine and Müller cells and in the axon terminals of type 2 cone bipolar cells. These results are, to some extent, in agreement with those of previous studies that have reported the presence of MAGL in the OPL (rod spherules and cone pedicles), IPL and GCL (Hu et al., 2010). We believe that the expression reported by Hu et al. (2010) might reflect the presence of MAGL in Müller cells. Müller cell bodies sit in the INL and project processes in either direction to the outer and inner limiting membranes. Therefore, somata or processes of these cells are present in every retinal cell layer and might be responsible for the localization of MAGL reported by Hu et al. (2010) in adult animals.

#### MAGL localization in the IPL

MAGL was also intensely expressed in the IPL. MAGL was present in the axon terminals of type 2 OFF cone bipolar cells, but not in type 8 ON cone bipolar cells or in rod bipolar cells. However, MAGL was not restricted to type 2 cone bipolar cells. On the basis of the position of the MAGL expression in the IPL, we hypothesize that MAGL might be present in the axon terminals of type 3 or 4 OFF cone bipolar cells. Additionally, we showed that MAGL was not detectable in the dendrites of amacrine or ganglion cells.

#### Functional considerations

Because eCBs are lipophilic ligands that are released and degraded near their sites of action, one would predict that DAGLα and MAGL would have similar distributions. Obviously, DAGLα and MAGL do not share a similar distribution; DAGLα was expressed in nearly every retinal cell type, and MAGL was only expressed in amacrine and Müller cells. However, because Müller cells stretch radially across the thickness of the retina and insinuate themselves between the cell bodies of neurons in the nuclear layers and envelop groups of neural processes in the plexiform layers, DAGLα and MAGL are nearly co-expressed in every retinal cell layer.

DAGLα and MAGL are often near or in the same cell types as CB1R and CB2R. CB1R is present in cones, horizontal, bipolar, amacrine and ganglion cells in the developing rat retina (Zabouri et al., 2011a). CB2R is present in cone and rod photoreceptors and horizontal, bipolar, amacrine and ganglion cells in the adult mouse retina (Cécyre et al., 2013). Taken together, these results suggest that, in the retina, eCBs, such as 2-AG, are expressed closely to cannabinoid receptors and might be implicated in the regulation of the maturation of the retina. Because eCBs are known to participate in developmental processes, such as neurogenesis, axon guidance and synaptogenesis, they might play an essential role in the structural and functional development of the retina.

The very low levels of protein content of MAGL in the retina from birth to P11 were surprising given the importance of MAGL in 2-AG hydrolysis. Because MAGL is responsible for approximately 85% of the hydrolysis of 2-AG, it is possible that the enzymes ABHD6 and ABHD12 mediate 2-AG hydrolysis in the first days of development when MAGL is absent.

In conclusion, this study revealed the presence of DAGLα and MAGL in the retinal cells of developing and adult rats. We propose that retinal 2-AG levels increase from birth to P11 and are then regularized in adulthood. DAGLα, MAGL, CB1R and CB2R proteins are present in young and adult animals, which suggests that the eCB system might play an important role in the development and function of the retina.

## Author contributions

All authors had full access to all the data in the study and take responsibility for the integrity of the data and the accuracy of the data analysis. Study concept and design: Bruno Cécyre, Christian Casanova, Jean-François Bouchard. Acquisition of data: Bruno Cécyre, Marjorie Monette, Liza Beudjekian. Analysis and interpretation of data: Bruno Cécyre, Marjorie Monette, Liza Beudjekian, Christian Casanova, Jean-François Bouchard. Drafting of the manuscript: Bruno Cécyre, Christian Casanova, Jean-François Bouchard. Statistical analysis: Bruno Cécyre. Obtained funding: Christian Casanova, Jean-François Bouchard.

## Conflict of interest statement

The authors declare that the research was conducted in the absence of any commercial or financial relationships that could be construed as a potential conflict of interest.
